# Flame Retardancy of Bio-Based Polyurethanes: Opportunities and Challenges

**DOI:** 10.3390/polym12061234

**Published:** 2020-05-29

**Authors:** Henri Vahabi, Hadi Rastin, Elnaz Movahedifar, Karina Antoun, Nicolas Brosse, Mohammad Reza Saeb

**Affiliations:** 1 Université de Lorraine, CentraleSupélec, LMOPS, F-57000 Metz, France; 2 School of Chemical Engineering, College of Engineering, University of Tehran, Tehran 1417466191, Iran; hadirastin88@gmail.com; 3 Department of Polymer Engineering, Amirkabir University of Technology-Mahshahr Campus, Mahshahr 424, Iran; el.movahedifar@gmail.com; 4 Université de Lorraine, INRAE, LERMAB, F-54000 Nancy, France; karina.antoun@univ-lorraine.fr (K.A.); Nicolas.Brosse@univ-lorraine.fr (N.B.); 5 Department of Resin and Additives, Institute for Color Science and Technology, Tehran 16765-654, Iran

**Keywords:** bio-based polyurethane, flame retardancy, flame retardants, renewable resources, sustainability

## Abstract

Sustainable polymers are emerging fast and have received much more attention in recent years compared to petro-sourced polymers. However, they inherently have low-quality properties, such as poor mechanical properties, and inadequate performance, such as high flammability. In general, two methods have been considered to tackle such drawbacks: (i) reinforcement of sustainable polymers with additives; and (ii) modification of chemical structure by architectural manipulation so as to modify polymers for advanced applications. Development and management of bio-based polyurethanes with flame-retardant properties have been at the core of attention in recent years. Bio-based polyurethanes are currently prepared from renewable, bio-based sources such as vegetable oils. They are used in a wide range of applications including coatings and foams. However, they are highly flammable, and their further development is dependent on their flame retardancy. The aim of the present review is to investigate recent advances in the development of flame-retardant bio-based polyurethanes. Chemical structures of bio-based flame-retardant polyurethanes have been studied and explained from the point of view of flame retardancy. Moreover, various strategies for improving the flame retardancy of bio-based polyurethanes as well as reactive and additive flame-retardant solutions are discussed.

## 1. Introduction

Polyurethanes (PUs) are a class of polymers with versatile properties known for their variable chemical structure and flexibility in their synthesis routes [1,2]. The wide range of possibilities in chemical manipulation of PU backbone offers a powerful tool for tailoring their ultimate properties [3]. As a result of such varieties in microstructure, PUs have been widely considered in numerous sectors such as the shoes market, high-performance adhesives, tires, construction, high-resilience foams, rigid foam insulation panels, biomedical applications, electrical insulations, and coatings [4,5,6,7]. According to a recent report, the global PU market with a compound annual growth rate (CGAR) of 6.0% is estimated to reach USD 88 million by 2026 [8,9].

From a chemistry standpoint, PUs are identified as organic polymers with urethane links, which are the result of a chemical reaction between isocyanates and polyols. The versatility of their chemistry allows the obtaining of thermoset or thermoplastic PUs [9]. In the synthesis of conventional PUs, polyols and isocyanates are usually derived from petrochemical sources. Over recent decades, growing concerns about global warming resulting from human activity, depletion of fossil resources, and fluctuating oil price has prompted debate among researchers exploring bio-based resources to replace starting chemicals with green, sustainable, and renewable sources [10,11,12,13,14]. Thus, biopolymers derived from biological and renewable sources have attracted the attention of researchers due to their sustainability, abundant availability, and eco-friendliness [4].

The development of bio-based PUs using bio-based/renewable isocyanates and/or polyols has also attracted the interest of academic and industrial researchers [15,16,17,18,19]; accordingly, several review papers have been published that rely on such a necessity [20,21,22]. Moreover, the development of isocyanate-free PU-based materials has been recognized as an efficient approach to avoid the toxicity issues associated with the use of isocyanates. Several companies currently produce and market bio-based PUs, such as Dow Chemical [23], BASF [24], Huntsman [25], Rampf Holding [26], Alberdingk [27], Lubrizol [28], Mitsui Chemicals [29], Woodbridge Foam [30], Croda [31], etc. The overall bio-based PUs market was 1634 tons in 2013 and is expected to reach 2546.6 tons by 2020 [32,33].

Bio-based PUs are highly flammable, which obstructs their broad development [34]. There are various solutions for improving the flame retardancy of PUs, including reactive and additive methods, and several review papers have already been published in this regard [35,36,37]. However, these reviews are related to the flame retardancy of petroleum-based PUs, and, to the best of our knowledge, there has been no review of the flame retardancy of bio-based PUs. In the present review, first the synthesis and origin of bio-based PUs are briefly described. Then, the state-of-the-art strategies used in the improvement of flame retardancy of bio-based PUs are reviewed and discussed.

## 2. Synthesis of Bio-Based PUs

All PUs contain the urethane function (–NH–(C=O)–O–), which is synthesized by the reaction between an isocyanate (–NCO) group and an alcohol (–OH). Polyols are a class of organic molecules with multiple hydroxyl functional groups that are chemically potent to react with isocyanates. These molecules can be obtained from renewable sources. The chemistry of bio-based PUs has been described in several review papers [4]. Table 1 gives the origin of some bio-based non-exhaustive molecules used in the production of bio-based PUs.

A promising approach with common “green polyols” is based on the use of lignin [38]. Lignin is one of the most abundant renewable polymers, produced in large amounts by the pulp industries and emerging bio-refineries. Attention towards lignin has significantly increased, because this attractive biopolymer is rich in low-cost renewable carbon for the production of bio-based materials [39,40]. Since lignin contains aliphatic and phenolic hydroxyl groups, it has been examined as a low-cost abundant macropolyol for the preparation of lignin-based PUs [41,42,43]. In addition, the natural properties of lignin and its chemical features contribute to reduce the consumption of isocyanates and improve the flame resistance of PU materials [44,45]. Indeed, due to its aromatic structure, lignin has an inherent ability to generate stable char during combustion [46].

**Figure 1:**
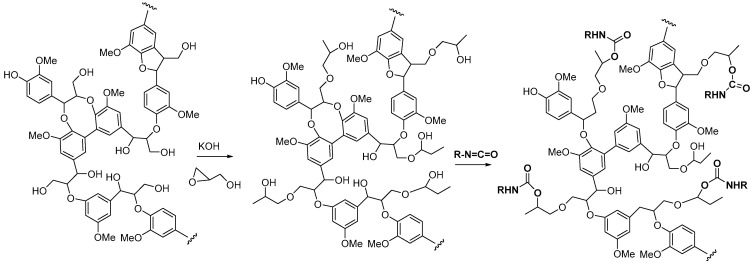
Oxypropylation of lignin for the production of lignin-based PU.

The word “lignin” does not refer to a homogeneous polymer, but frequently to a complex mixture, highly variable as a function of the plant taxonomy and the delignification process used, with various ranges of molar masses and diverse distributions of functional groups [40,47]. As a result, due to the poor quality and high variability of industrial lignins as well as their low chemical reactivity, their incorporation into PU foam formulations can be done at low contents [48,49]. Incorporation of unmodified lignin over 30 wt.% has been reported to produce brittle foams with soft and hard segments, which strongly affects the mechanical properties of the resulting PU, producing brittle and friable foams [50]. Chemical functionalization routes have been described to address these issues. The increased reactivity of lignin with isocyanates has been extensively studied by demethylation of methoxyl groups in the lignin structure or by the introduction of primary (and secondary) alcohols through the grafting of hydroxylated chains onto various sites of the lignin polymer; see Figure 1. The addition of chain extenders such as castor oil, polyethylene or polypropylene glycol, and butanediol has also been examined [51].

Among the bio-based molecules used in synthesis of PUs are vegetable oils, which are widely explored [4]. These molecules can be converted to different monomers similar to petroleum-based ones as a greener alternative. Vegetable oils are predominantly composed of triglycerides with three long fatty acid chains. The length of fatty acid and number and the location of double bonds in the chemical structure of triglycerides vary for different vegetable oils, which determine their chemical and physical properties [54]. Unsaturated double bonds along fatty acids are not reactive enough in free-radical polymerization. Thus, modification of existing double bonds is an inevitable step to incorporate the desired functional groups. Another alternative to achieve more reactive monomers is the modification of ester groups.

**Figure 2:**
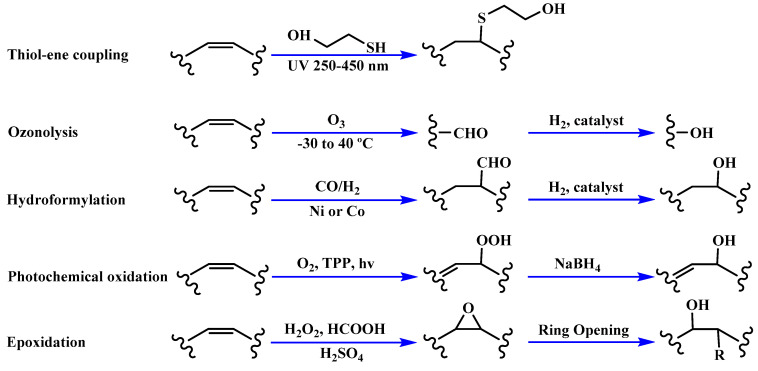
Synthesis of bio-based polyols derived from vegetable oils [77].

Polyols can be obtained from various synthesis methods such as converting double bonds to hydroxyl groups in vegetable oils. Overall, multiple pathways have been reported in the synthesis of polyols from vegetable oils including thiol-ene coupling reaction, ozonolysis, hydroformylation, photochemical oxidation, and epoxidation, as illustrated in Figure 2. In the thiol-ene reaction, unsaturated double bonds react with thiol section of 2-mercaptoethanol in a mild condition to generate a hydroxyl group [71]. Ozonolysis is another approach to generate polyol from vegetable oils. Unsaturated double bonds along the fatty acids are oxidized first to produce aldehyde groups followed by a reduction to hydroxyl groups [71]. In hydroformylation, aldehyde groups are generated first through the reaction of double bonds with hydrogen and carbon monoxide, then are converted to hydroxyl group [72]. Another possible route to yield hydroxyl group along the fatty chain is photochemical oxidation, during which fatty acids are oxidized with sunlight source in a rich oxygen environment once exposed to high-pressure sodium-vapor lamp and tetraphenyl porphyrin as a sensitizer to produce allylic hydroperoxide [71]. Following that, hydroperoxide can be converted into hydroxyl groups via reduction with sodium borohydride. Finally, epoxidation is the main approach to derive polyols from vegetable oils followed by ring-opening reactions. Various ring-opening agents can be used to convert epoxy into OH groups such as water, alcohol, acids, and amines [73,74]. Multi-isocyanate is another building block of PU. The most widely used multi-isocyanates for the production of PU are 2,4-toluene diisocyanate, 2,6-toluene diisocyanate, 4,4-diphenylmethane diisocyanate, 1,6-hexamethyl diisocyanate, xylene diisocyanate, and isophorone diisocyanate, which are the product of a chemical reaction between gaseous phosgene with amines [75]. However, growing concern associated with the highly toxic nature of the precursors involved in the synthesis of multi-isocyanate dictates the use of an alternative eco-friendly approach [3,11]. Multi-isocyanate can be derived from vegetable oils and their derivatives, increasing the biomass content of PUs. A chemical reaction between unsaturated double bonds in the soybean oil and iodine isocyanate yields an isocyanate-containing soybean oil with an average number of three isocyanate groups per triglyceride [76,77].

**Figure 3:**
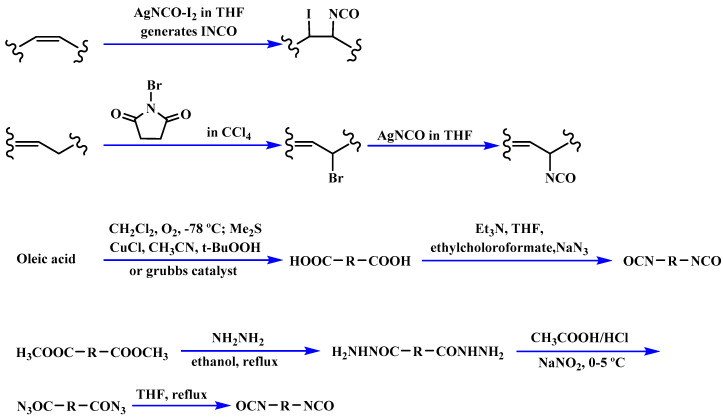
Synthesis of bio-based multi-isocyanate derived from vegetable oils [77].

The bromination of soybean oil followed by reaction with AgNCO leads to the preparation of multi-isocyanate, in which the substitution degree of Br with NCO groups depends on the concentration of AgNCO [75], Figure 3 [77]. However, PUs prepared from isocyanate-containing soybean oil show weak mechanical strength. Another possible option to achieve bio-based multi-isocyanate is via the modification of fatty acids.

In a study conducted by Hojabri et al. [78], Curtius rearrangement was used to prepare linear bio-based 1,16-diisocyanatohexadec-8-ene (HDEDI) and 1,7-heptamethylene diisocyanate (HPMDI) starting from oleic acid fatty acid. PU synthesized from HDEDI displays higher mechanical strength compared to petroleum-based 1,6-hexamethylene diisocyanate, whereas the PU prepared from the HPMDI shows comparable strength due to the longer alkane chains. In another method, castor oil derivatives have been used to synthesize novel bio-based isocyanate and mixed with commercially available and bio-based polyols [79]. As previously mentioned, all methods used in the preparation of bio-based PUs have already been reviewed [4]; therefore, our focus will be placed on the flame retardancy of bio-based PUs.

## 3. Flame-Retardant Bio-Based PUs

Generally, two approaches have been applied to endow flame retardancy to bio-based PU structures: (i) the addition of flame-retardant additive to the bulk of polymer through melt processing and physical incorporation; and (ii) the addition of flame-retardant elements into the PU chains by reactive molecules and chemical incorporation. Herein, these methods are reviewed in two sections: additive and reactive solutions.

### 3.1. Flame Retardant as “Additive” in Bio-Based PUs

In the additive method, flame retardant, as a component of formulation and generally in the form of powder, is combined with PUs during the synthesis of the matrix to impart flame retardancy. Therefore, there is no chemical bond between the PU chains and the flame retardant. Generally, to improve flame retardancy, some parameters should be controlled in the formulation of flame-retardant PUs such as the size, loading percentage, and dispersion state of the flame retardant. Both organic and inorganic additives have been used in bio-based PUs to improve flame retardancy, Table 2.

Agrawal et al. [80] investigated the physical incorporation of zirconia and alumina fillers into the rigid PU foams derived from modified castor oil. The aforementioned fillers have been incorporated into the PU up to 10 wt.%. Overall, based on thermogravimetric analysis (TGA), higher thermal stability was obtained in the presence of zirconia. The authors explained the results by the role of zirconia as a cross-linker for PU backbone, as well as a better dispersion of this filler compared with alumina. In cone calorimetry, the presence of alumina and zirconia at 6 wt.% showed the best results by reduction in the peak of heat release (pHRR) from 118 for unfilled PU to 84 and 94 kW/m^2^ for alumina and zirconia incorporated PUs, respectively. At higher loading percentage, the performance decreases, which is explained by the agglomeration of particles and deterioration of the insulating effect during combustion. In another paper, these authors studied the flame retardancy of the same bio-based PU in the presence of feldspar and kaolinite clay [81]. A similar approach to that of the first paper was applied in their investigation. The results showed a similar performance of these clays in terms of flame retardancy by the physical barrier action in the condensed phase.

Ranaweera et al. [82] synthesized a bio-based polyol derived from limonene, extracted from orange peel. Dimethyl methyl phosphonate (DMMP), 2 parts by weight, has been used and blended as flame retardant into the limonene-based polyol and isocyanate to prepare flame-retardant PU foam. The authors performed horizontal burning test and observed a reduction in burning time by 83% compared to the neat sample. However, the incorporation of DMMP reduced the mechanical properties of the PU foams.

Ramanujam et al. [83] studied a bio-based PU based on corn oil and served DMMP as additive to improve the flame retardancy of PU foam. It was found that burning time, in horizontal burning test, of the flame-retardant PUs progressively declined upon increase of DMMP loading in the formulation. They succeeded in reducing the burning time from 115 s to 3.5 s by the incorporation of 5.5 wt.% of DMMP, i.e., 1.94 wt.% phosphorus in the material. A cone calorimetry test was also performed; however, the reduction in pHRR was not significant in the presence of DMMP.

Zhang et al. [84] synthesized a new flame retardant via the reaction of benzaldehyde, aniline, and 9,10-dihydro-9-oxa-10-phosphaphenanthrene-10-oxide (DOPO-BA). The obtained flame retardant has been incorporated into rosin-based rigid PU foam. The incorporation of 20 wt.% DOPO-BA led to an increase in the limiting oxygen index (LOI) value from 20.1% to 28.1%. The authors also reported a linear dependence of LOI on the DOPO-BA content (from 5 to 20 wt.%). In cone calorimetry, there was no significant effect on pHRR in the presence of DOPO-BA. However, the authors reported a significant decrease in the total smoke release, while a rise in the char residue content, and deducted a condensed phase mechanism.

Gao et al. [85] investigated the combination of expandable graphite (EG) and melamine polyphosphate (MPP) on the flame retardancy of a rosin bio-based PU. They found that the combination of these flame retardants at 10 parts per hundred of polyol by weight (php) was efficient in increasing the LOI from 21 to 26.5. Then, the authors evaluated the effect of layered double hydroxide (LDH) on this combination in terms of flame retardancy. The results revealed that the combination of 3 php LDH with 10 php EG and 10 php MPP led to increase in the LOI to 28. However, this combination was not as efficient as results collected in the cone calorimetry test, which was demonstrated by the similarity between the heat release rate (HRR) curves of EG/MPP and EG/MPP/LDH samples.

The synthesis of cashew Mannich polyol has been reported by Gandhi et al. [86]. The PU prepared by this polyol has been physically flame retarded with the incorporation of various fillers. They reported that the incorporation of 10 wt.% polyvinyl chloride (PVC) powder or Sb_2_O_3_ led to an increase in LOI. However, the same quantity of fly ash, ceramic, or Teflon as filler decreased the LOI value. Agrawal and Kaur [87] incorporated nano-CaCO_3_ in a castor oil bio-based PU to enhance its flame retardancy. The addition of 8 wt.% nano-CaCO_3_ decreased the pHRR in the cone calorimetry test to 93 kW/m² against 118 kW/m² for neat bio-based PU.

### 3.2. “Reactive” Flame Retardants in Bio-Based PUs

Generally, reactive flame retardants display higher thermal stability compared to additives, due to their chemical bonding with polymer chains. Unlike additive flame retardant, reactive flame retardants do not migrate under ageing conditions and hence provide flame retardancy over time [88,89]. Moreover, incorporation of flame retardant as the reactive part in the polymer structure avoids the deterioration of mechanical properties due to enhanced dispersion of flame retardant throughout the polymer chains [90]. Overall, it is possible to classify the reactive flame retardants used in bio-based PUs in different ways, including the function of the used flame-retardant element (phosphorus, nitrogen, etc. -based flame retardants). However, most research works focus on phosphorus reactive flame retardants; see Table 3. It is worth mentioning that in the present review, the research works exploring the reactive halogenated [91] solutions were not considered.

Bhoyate et al. [92] synthesized a phosphorus bio-based polyol through the reaction of diethyl allyl phosphonate (DEAP) and thioglycerol (TG), and mixed it with different bio-based polyols including soybean, orange peel, and castor oil-based polyol. They succeeded in incorporating 1.5 wt.% phosphorus into the PU foam. In terms of flame retardancy performance, the horizontal burning test revealed a substantial fall in extinguishing time from 94 s to 1.7 with 1.5 wt.% reactive phosphorus. In the cone calorimeter test, the pHRR decreased from 313 kW/m^2^ to 158 kW/m^2^. The authors explained this improvement by formation of phosphinic acid during the combustion and formation of a protective char layer on the surface of the foam. Bhoyate et al. [93] also examined the effect of a bio-based mercaptenized castor oil polyol, modified with DEAP in the PU. Surprisingly, similar results have been obtained in cone calorimetry tests. In another work, Bhoyate et al. [94] investigated the flame retardancy of a bio-based polyol derived from limonene and chemically modified by phenyl phosphonic acid. They reported that chemical incorporation of 1.5 wt. % of phosphorus can reduce the self-extinguishing time of the foam from 81 s to 11.2 s. In the cone calorimeter, pHRR and the total heat release (THR) have been decreased 68.6% and 23.4%, respectively. A higher graphitization of the char formed in the presence of phosphorus, and higher barrier quality prevented the release of combustible volatility, which is the reason flame retardancy improved. 

**Figure 4:**
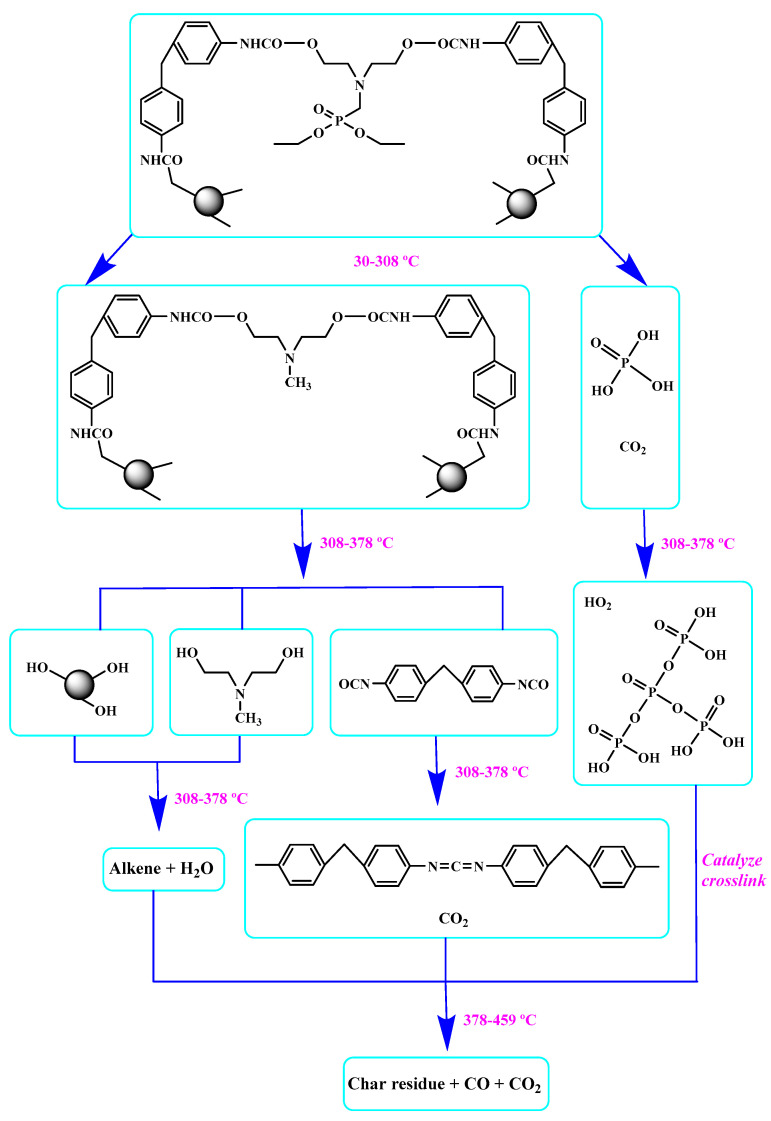
Mechanism of thermal degradation for flame retardant (FR bio-based PU proposed by Ding el al. [96].

Ding el al. [95] synthesized a novel bio-based polyol derived from castor oil containing both phosphorus and nitrogen. In cone calorimetry, the pHRR has been decreased by 51.5% with respect to the non-flame-retardant PU. The authors explained the improvement of flame retardancy by the synergy between phosphorus and nitrogen, which led to the formation of high char content. The char formed played the role of heat and mass barriers between the condensed and gas phases. In another work, the same research team explored [96] the flammability of a PU obtained from a bio-based polyol containing both nitrogen and phosphorus; see Table 3. The results of the flame test in cone calorimetry showed a significant decrease in the pHRR and THR, respectively by 48% and 14% compared with the neat bio-based PU. The authors performed a series of analyses including TGA, TGA coupled with Fourier-transform infrared (FTIR) spectroscopy, char analysis by FTIR, and X-ray photoelectron spectroscopy (XPS) and suggested a mechanism for thermal degradation of their bio-based PU; see Figure 4. The mechanism of thermal degradation proposed by Ding et al. [89] suggested a first step of degradation from room temperature to 308 °C consisting of P–O–C and P–C bond breakup, which led to the formation of phosphoric acid, H_2_O, and CO_2_. Then, from 308 °C to 378 °C, the degradation of urethane groups led to the formation of isocyanate and hydroxyl compounds.

From this point, several reactions simultaneously happen with the formation of carbodiimides and hydrocarbon compounds. The phosphoric acid forms polyphosphoric acid, which has a catalytic action in crosslinking reaction and char formation. The char obtained in the presence of phosphorus and nitrogen as well as aromatic compounds has a higher thermal stability and has a barrier effect at high temperatures. The same research group also worked on a new bio-based polyol containing 9,10-dihydro-9-oxa-10- phosphaphenanthrene-10-oxide (DOPO) and nitrogen to synthesize PU [97]. Then, they blended this flame-retardant polyol with the obtained polyol from their previous study (BHAPE). The flame retardancy of the obtained PU has been studied using cone calorimeter tests. The pHRR decreased from 359 kW/m^2^ for neat PU to 139 kW/m^2^ in the case of flame-retarded PU. The THR value has also been decreased from 21.2 MJ/m^2^ to 9.1 MJ/m^2^. Once again, the authors analyzed the obtained char and evolved gases in TGA by various characterization methods to propose a mechanism for thermal degradation. In the proposed mechanism, the degradation of the P–O–C, P–N, and P–C links was first observed. Then, free-radical scavengers were formed that inhibited the free-radical chain reactions. At higher temperatures, the aliphatic and aromatic compounds were formed and then in the presence of polyphosphoric acid, polyaromatic char structure was formed. Thus, according to the authors, the flame retardant acted in both gas and condensed phases. Özşeker et al. [98] synthesized a novel bio-based polyol containing phosphorus through a ring-opening reaction of epoxidized soybean oil (ESBO) and vinyl phosphonic acid (VPA). The authors only reported the LOI test and mentioned that the LOI value of flame-retardant PU was 26.4. The same research group also reported the synthesis of two bio-based PUs containing reactive 3-mercaptopropyltrimethoxysilane (MPTMS), and 3-aminopropyltriethoxysilane (APTES) [99]. They also studied the effect of addition of the phosphorus groups during the synthesis via the chain extenders. Flame retardancy properties have been investigated using UL-94 and LOI tests. The obtained PUs containing MPTMS and APTES have been rated V-1 in UL-94 with LOI of 23.6 and 22.8. The authors reported that the reactive addition of phosphorus via chain extender led to an increase in LOI to 26.3 and V-0 rate in UL-94. However, the mechanism of flame retardancy has not been explained. Mestry et al. [100] developed a cardanol-based PU for coating applications by integrating phenylphosphonic dichloride (PPDC) and dichlorodimethylsilane (DCDMS) on polyol. The best sample in terms of flame retardancy was the system containing 50:50 composition of P:Si with LOI equal to 29 and V0 in UL-94 test. This behavior has been explained by the char-forming character of phosphorus and char-promoting character of Si, which aided the condensed phase action. Heinen et al. [101] prepared a phosphorylated polyol through reaction of epoxidized soybean oil with phosphoric acid. LOI test showed that the value of LOI increased by increasing the content of phosphorus. The value of LOI was 21.8 for PU containing 0.86 wt.% phosphorus. Patel et al. [102] integrated phosphorus molecules on bio-based gallic acid (GA) and prepared a GA-based phosphorus curing agent for synthesis of PU. They reported that by addition of 15%mol of the phosphorus GA to the formulation of PU, the V0 in UL-94 and 27 value in the LOI were obtained against V2 and 21 for the neat PU.

The addition of lignin or the modified lignin into the PU was generally described to improve mechanical properties and to increase the decomposition temperature of the resulting material. To further increase the fire performance of lignin-based PUs, different strategies have been employed. Lu et al. [45] have shown that 15–20% lignosulfonate loading increases thermal stability of rigid PU foams because of a higher char production. The addition of (ammonium polyphosphate) APP to the lignosulfonate further increases the fire performance. The combination of lignin, clays, and a phosphorus have been studied for the production of flexible [103] and rigid [104] PU foams. In all these studies, a decrease in the HRR during combustion associated with a limitation of flame propagation was observed through the formation of inert gases, and a protective char layer during combustion.

The chemical functionalization of lignin has also been examined by grafting nitrogen and phosphorus via a three-step reaction to improve its flame retardancy [105]. It has been shown that the modified lignins exhibited a much higher thermal stability reducing the pHRR, the THR, and the smoke production rate during combustion. Zhang et al. [106] reported the synthesis of a lignin-based flame retardant through the reaction between organosolv lignin and 9,10-dihydro-9-oxa-10-phosphaphenanthrene-10-oxide (DOPO). Lignin-based PUs displaying good flame-retardant properties have been synthesized by the reaction between modified lignin, hexamethylene diisocyanate, and polyethylene glycol. Modified lignin containing chemically grafted phosphorus and nitrogen by liquefaction–esterification–salification has been used to prepare PU foam. Compared to pure PU the lignin-modified PU foam showed a negligible negative influence on the morphology and mechanical properties and displayed an excellent performance of thermal stability, char residue formation, self-extinguishment, and inhibition from melt-dripping and smoke generation. According to the authors, these properties were fueled by the rigid aromatic structure of lignin and the covalent linkages between lignin-based phosphate melamine compound and the polymer matrix [107]. Thus, engineering of flame retardancy of bio-based PU requires knowledge of chemistry.

## 4. Conclusions

Bio-based PUs are one of the most attractive biopolymers, and considerable attention has been devoted to their development due to sustainability issues. Even if bio-based PUs have a high versatility in properties due to the variety of their synthesis routes, they suffer from high flammability. The aim of the present review was to summarize the recent advances on the flame retardancy of bio-based PUs including additive and reactive methods. The monitoring of the literature showed that there are few papers on the flame retardancy of bio-based PUs. It has also been revealed that most reactive solutions consist of the modification of polyols with phosphorus agents. However, the reactive flame-retardant solutions are not always bio-based. This point should be considered in future research with fully bio-based PUs. One solution could be the use of lignin [38], as presented in some papers, to enhance flame retardancy. Moreover, the additive flame-retardant solution should be meticulously selected, not only in terms of flame retardancy efficiency, but also other properties that should be monitored and optimized. For example, incorporation of additives can modify the size of pores or increase the thermal conductivity in foams, which are the key properties needed for PUs. Moreover, PUs widely contribute to building and construction; therefore, their ageing should additionally be taken into account in formulations. The ageing of flame-retardant PUs should also be considered to evaluate the effect of ageing on PU in the presence of flame retardant. Furthermore, the effect of ageing on the efficiency of flame retardant during its lifetime should be considered in designs. For both additive and reactive solutions, the effect of flame-retardant presence on the mechanical properties of bio-based PUs should be particularly monitored.

## Figures and Tables

**Table 1:** Information extracted from the literature on bio-based PUs.

Matrix	Bio-Based	Property Studied	Application	Ref.
PU foam	Coffee grounds waste-based polyol	Mechanical, Thermomechanical, Thermal, Physical properties	Acoustic insulation and aeronautics	[52]
Rigid PU foam	Palm oil-based polyester polyol	Physical, Thermal, Mechanical properties	Foam	[53]
PU foam	Soy-castor oil-based polyol	Physical, Thermal, Mechanical properties	PU foams with thermal stability and high thermal conductivity	[54]
PU	Lignin-oleic acid-based polyol	Thermomechanical, thermal, Mechanical, rheological properties	Building and automotive industries	[55]
Hyperbranched PU	Mesua ferrea L. seed oil-based monoglyceride	Thermal, Mechanical properties, Chemical resistance	High-performance coating materials	[56]
Hyperbranched PU	Sunflower oil-based fatty acid	Mechanical, Thermal, properties, Chemical resistance	Scaffold for Tissue Engineering	[57]
Rigid PU foam	Cooking oil bio-based polyols	Thermoelectric, Thermal, Mechanical properties	Open-cell PU foams for applications in construction industry	[58]
Glass fiber reinforced PU	Soy oil-based polyol	Thermal, Mechanical, Thermomechanical properties	Seat pans, sunshades, door panels, package trays, and truck box panels	[59]
PU elastomer/cellulose nanowhisker composite	Castor oil-based polyol	Thermal, Mechanical, Thermomechanical properties	Composite with mechanical properties reinforced	[60]
PU	Vegetable oil-based polyols from refined canola and sunflower oils, and camelina, Linola^®^ 2090 flax and NuLin^®^ 50 flax crude oils	Thermal, Mechanical, Thermomechanical, Surface properties, Swelling of PU networks and crosslinking density	High-solid PU coatings	[61]
Waterborne PU	Vegetable oil-based polyols (peanut, corn, soybean, and linseed)	Thermal, mechanical, Thermomechanical properties	PU films	[62]
Poly(ester urethane) metallohybrid	Linseed oil-based polyol	Thermal, Coating properties, Physico-chemical characterization	Antibacterial self-sterilizing protective coatings	[63]
PU	Vegetable oil-based dimer fatty acid	Thermal, Surface, Coating properties, Water uptake, Chemical resistance	Transparent PU films and coatings	[64]
Polyurethane/graphene oxide	Soybean-castor oil fatty acid-based polyol	Thermal, Thermomechanical, Mechanical properties	Composite with mechanical properties reinforced	[65]
PU/siloxane hybrid	Silanized castor oil-based functional polyol	Thermal, Mechanical, Surface properties, Water resistance	Water repellent coatings	[66]
Crude glycerol-based waterborne PU	Soy meal protein and crude glycerol	Mechanical, Thermal properties, Water resistance	Hydrophobic coating, Packaging application	[67]
Poly(urethane fatty amides)	Neem oil fatty amide	Thermal, Coating properties, Chemical and Corrosion resistances	Industrial coating	[68]
Thermoset PU	Canola oil-based poly (ether ester) polyol	Thermal, Thermomechanical Adhesive properties, Chemical resistance	Adhesive	[69]
Rigid PU Foam	Soybean oil-based Polyol	Mechanical, Thermal properties, Swelling	automotive interior, office furniture	[70]

**Table 2:** Summary of “additive” flame-retardant bio-based PUs (Numbers 1 to 4 correspond to the “inorganic”, while numbers 5 to 7 correspond to the “organic”, and number 8 corresponds to both “inorganic and organic” flame-retardant additives).

N°	Type of PU	Bio-Based Molecule Used in Synthesis of PU	Flame Retardant	Ref.
1	Rigid PU foams	Castor oil-based polyol	Zirconia and alumina	[80]
2	Rigid PU foams	Castor oil-based polyol	Nano-CaCO_3_	[87]
3	Rigid PU foams	Castor oil-based polyol	Feldspar and kaolinite	[81]
4	Rigid PU foams	Rosin-based polyol	Expandable graphite and melamine polyphosphate, and layered double hydroxide	[85]
5	Rigid PU foams	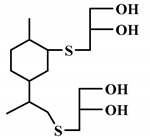	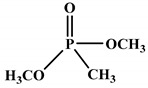	[82]
6	PU foams	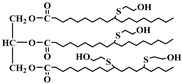	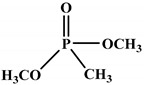	[83]
7	Rigid PU foams	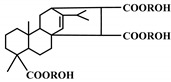	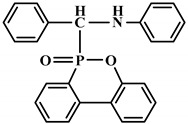	[84]
8	Rigid PU foams	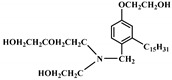	Fly ash, ceramic, Teflon, antimony trioxide, polyvinyl chloride powder	[86]

**Table 3:** Summary of reported works on bio-based PUs using “reactive” flame retardants (numbers 1 to 6 correspond to the phosphorus-containing flame retardant, numbers 7 to 9 correspond to the phosphorus and nitrogen-containing flame retardant, number 10 corresponds to the silicon-containing flame retardant, and number 11 corresponds to the silicon- and phosphorus-containing flame retardant).

N°	Type of PU	Bio-Based Molecule Used in Synthesis of PU	Flame Retardant	Flame Retardant Polyol	Ref.
1	Rigid PU foams	Soybean, orange peel, and castor oil	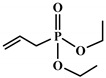	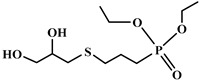	[92]
2	Rigid PU foams	Castor oil	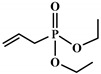	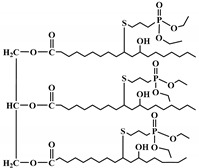	[93]
3	Rigid PU foams	Limonene	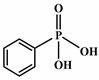	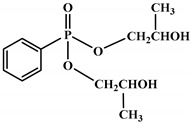	[94]
4	Rigid PU foams	Soybean oil	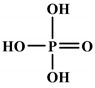	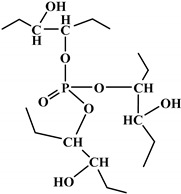	[101]
5	PU foams	Soybean oils	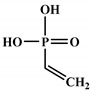	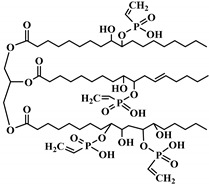	[98]
6	PU coating	Gallic acid	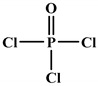	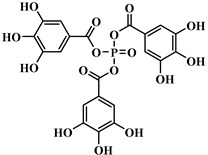	[102]
7	PU sealant	Ricinoleic acid (obtained from castor oil)	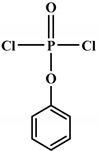	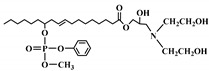	[95]
8	Flexible PU sealant	Castor oil	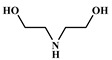 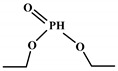	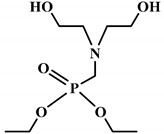	[96]
9	Flexible PU	Castor oil	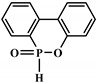 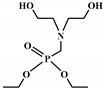	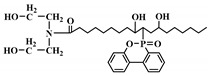	[97]
10	PU foams	Soybean oils	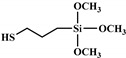 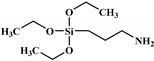	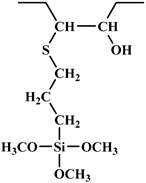 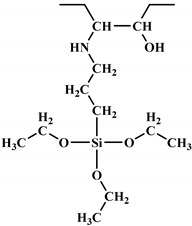	[99]
11	PU coating film	Cardanol	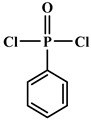 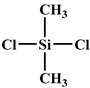	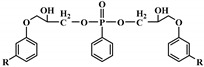 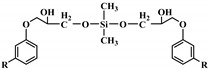	[100]
